# Quantitative Immobilization of Phthalocyanine onto Bacterial Cellulose for Construction of a High-Performance Catalytic Membrane Reactor

**DOI:** 10.3390/ma10070846

**Published:** 2017-07-24

**Authors:** Shiliang Chen, Qiaoling Teng

**Affiliations:** Qianjiang College, Hangzhou Normal University, Hangzhou 310012, China; tengql1996@sina.com

**Keywords:** bacterial cellulose, phthalocyanine, nanocomposite, catalytic membrane reactor

## Abstract

We report the fabrication of a tetra-amino cobalt (II) phthalocyanine (CoPc)-immobilized bacterial cellulose (BC) functional nanocomposite, CoPc@BC, by quantitative immobilization of CoPc onto a BC membrane. Lab-cultured BC was oxidized by NaIO_4_ to generate aldehyde groups on BC for the subsequent CoPc immobilization, the processing conditions were optimized by monitoring both the generated aldehyde content and the resulting CoPc loading. X-ray photoelectron spectroscopy (XPS) was employed to characterize the change of the element bonding environment during the functionalization processes. The CoPc@BC functional nanocomposite was utilized for the treatment of reactive red X-3B dye wastewater. The CoPc molecules in the CoPc@BC nanocomposite can function as an “antenna” to adsorb the target anionic dye molecules, the adsorption takes place both on the surface and in the interior of CoPc@BC. A catalytic membrane reactor (CMR) was assembled with the CoPc@BC nanocomposite, the performance of CMR was evaluated based on the catalytic oxidation behavior of reactive red X-3B, with H_2_O_2_ as an oxidant. Highly-reactive hydroxyl radical (OH) was involved in the catalytic oxidation process, as detected by electron paramagnetic resonance (EPR). Under optimal operating conditions of a flow rate of 6 mL/min, a reaction temperature of 50 °C, and an H_2_O_2_ concentration of 10 mmol/L, the decoloration rate of CMR was as high as 50 μmol⋅min^−1^⋅g^−1^.

## 1. Introduction

Metal phthalocyanine complexes (MPcs) are very attractive as catalysts not only because they are structurally related to porphyrin complexes, but also due to their accessibility from a preparation point of view, as well as their chemical and thermal stability [1,2,3,4]. In practical applications, MPcs are often adsorbed, covalently-anchored, grafted, and encapsulated onto or into solid supports [5,6,7,8,9,10,11], which can offer better catalytic activity, the possibility for rapid separation from the reaction media, and feasible catalyst recycling for a continuous reaction, thus overcoming the formation of inactive aggregates [12,13], recoverability, and reusability limitations associated with free MPcs. The property of the support has a primary importance on the catalytic performance of MPc, thus choosing appropriate supporting material for MPc immobilization is considered a major challenge. 

To improve the catalytic activity of MPc, a specific microenvironment provided by the support is essential. Several factors should be considered for the appropriate choice of the support: capacity to readily introduce functionality for anchoring, the degree of functionalization, the possible involvement with the reaction, the stability of the support under reaction conditions, and the availability of the support [1]. Among all of the supporting materials available for MPc immobilization, the bacterial cellulose (BC) membrane stands out due to several attractive characteristics [14,15,16,17]: it has a very large surface-to-volume ratio with easily tunable hydroxyls groups, thus, high MPc loading is, therefore, reasonably expected. It is produced in membrane form, the web-like network with a distinct tunnel and porous structure allowing the substrate to be readily accessible to reaction sites under very low diffusion resistance. In addition, BC is relatively stable under reaction conditions and is easily recoverable from the reaction media. 

Among various practically-applied catalytic reaction technologies, catalytic membrane reactors (CMR) are subject of intensive studies [18,19,20,21]. CMR are structured reactors combining, in a single unit, a membrane that controls mass transfers, and a catalyst providing chemical activity [22,23,24]. With the combination of catalyst-immobilization and membrane technology, favorable synergic effects could be reasonably expected: the membrane contributes to the adequate performance of the continuous process, which greatly increases the surface accessibility and contact area between the heterogeneous catalyst and the substrate within a fixed volume. As a result, the efficiency of the catalytic reaction is substantially improved. In particular, constructing cellulose-based high-performance CMR is a field of growing interest [25,26,27,28,29,30]. 

In the present work, a BC membrane was employed for the immobilization of the tetra-amino cobalt (II) phthalocyanine (CoPc) catalyst. The BC was pre-oxidized by NaIO_4_ to generate aldehyde groups, to which CoPc-containing amino groups can be attached through covalent bonding. The extent of oxidation is well controlled prior to CoPc immobilization. A high-performance CMR was assembled with the prepared functional nanomaterial, CoPc immobilized BC nanocomposite (CoPc@BC). With reactive red X-3B dye wastewater as the reaction model, the effects of operating parameters, that is, dye solution flow rate, operation temperature, and oxidant concentration on the performance of CMR, was investigated. The electron paramagnetic resonance (EPR) technique was employed to reveal the mechanism of the catalytic oxidation process. It is expected that this work may provide better understanding for MPc immobilization, BC functionalization, and further application of CMR in industry.

## 2. Materials and Methods

### 2.1. Materials and Reagents

*Acetobacter xylinum* was purchased from BeNa Culture Collection Co., Ltd. (category No. BNCC336985; Beijing, China). Reactive red X-3B (C.I. Reative Red 2, C_19_H_10_Cl_2_N_6_Na_2_O_7_S_2_, M.W.: 615.33) was purchased from Shanghai Chemical Reagent Factory (Shanghai, China). All other chemicals were of analytical grade and purchased from Sinopharm Chemical Reagent Co. Ltd. (Shanghai, China). Bacterial cellulose nanofibers were produced by cultivating the bacteria *Acetobacter xylinum* in a liquid culture medium containing 8.0 *w*/*v* % d-glucose, 1.0 *w*/*v* % yeast extract and 1.0 *v*/*v* % ethanol, as reported in the literature [31]. Tetra-amino cobalt (II) phthalocyanine (CoPc) was synthesized from 4-nitrophthalic acid, urea, and cobalt chloride hexahydrate according to a method described previously [32,33]. The spin-trapping agent, 5,5-dimethyl-1-pyrroline-*N*-oxide (DMPO), was purchased from Sigma Chemical Co. (Saint Louis, MO, USA). 

### 2.2. Preparation of CoPc@BC

**Figure 1:**
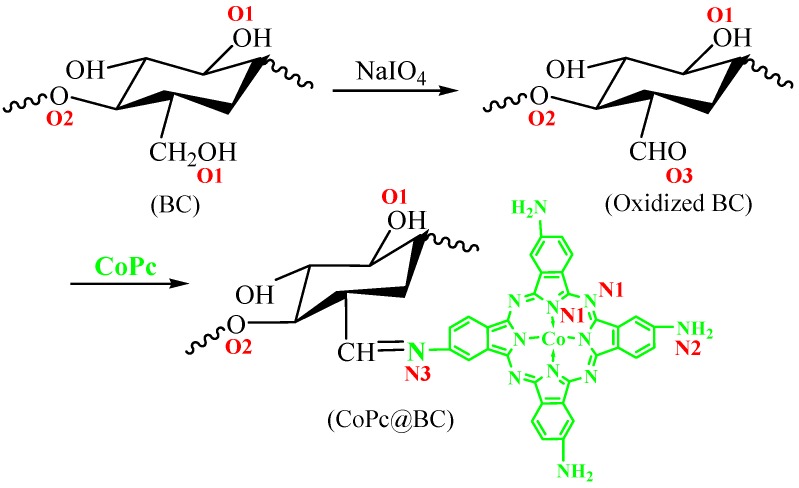
Synthesis route of CoPc@BC.

To prepare CoPc@BC, the following steps were taken. First, 20 mg of freeze-dried BC membrane was submerged into deionized water overnight, and then oxidized with NaIO_4_ solution at different concentrations elevated from 10 mmol/L to 50 mmol/L, the oxidation process was performed in a shaking water bath. The oxidized BC membrane containing aldehyde groups was thoroughly washed by ultrapure water and dried at 60 °C under vacuum. The dried membrane was suspended in a 200 μmol/L CoPc solution and reacted for 3 h at 25 °C, the product was washed several times with dimethylformamide (DMF) to eliminate residual CoPc, and then rinsed three times with ultrapure water. All the schematic functionalization processes are given in Figure 1. The influence of different reaction parameters, i.e., NaIO_4_ concentration, oxidation time, and reaction temperature, on the immobilized amount of CoPc was systematically studied. The aldehyde groups’ content on the oxidized BC was determined according to the literature, with minor modification [34,35]. An accurately-weighted 0.020 g oxidized BC was added to a 10 mL hydroxylamine hydrochloride solution at 50 °C for 2 h under stirring, the resulting mixture was titrated with 0.050 mol/L HCl solution with phenolphthalein as an indicator. A blank titration without oxidized BC was conducted under the same conditions. The aldehyde groups’ content of the oxidized BC was calculated as follows:

(1) $$\mathrm{Aldehyde}\;\mathrm{content}\;(\mathrm{wt}./\mathrm{wt}.\%)=\frac{0.050\;mol/L\times \left(V_{A}-V_{B}\right)mL\times 10^{-3}\times MW_{CO}}{0.020\;g}\times 100$$

where *V_A_* and *V_B_* are the consumed volumes of HCl solution for the blank and the sample, respectively. *MW_CO_* is the molecular weight of the carbonyl group. 

The cobalt content on CoPc@BC was measured using microwave-assisted digestion flame atomic absorption spectroscopy (Thermo Sollar M6, Thermo Fisher, Waltham, MA, USA), allowing the calculation of the amount of CoPc immobilized onto the BC. The CoPc loading was defined as the amount of CoPc (μmol) per gram of BC. Each value was the average of at least three separate experiments. 

### 2.3. Characterization

Field emission scanning electron microscopy (FESEM, Serion, FEI, Hillsboro, OR, USA) was applied to observe the morphologies of the nanofibers. The surface compositions of the BC, oxidized BC, and CoPc@BC were verified by attenuated total reflection Fourier transform infrared spectra (ATR/FT-IR). ATR/FT-IR spectra were acquired with a Vector 22 FTIR spectrometer (Brucker Optics, Faellanden, Switzerland) equipped with an ATR accessory (KRS-5 crystal, 45°) in the 4000–400 cm^−1^ range. The resolution was fixed at 4 cm^−1^ and a total of 32 scans were accumulated for each spectrum. The elemental compositions and chemical bonding of the BC, oxidized BC, and CoPc@BC were analyzed by X-ray photoelectron spectroscopy (XPS). XPS spectra were recorded on a Kratos Axis Ultra XPS system with Al (mono) Kα irradiation (hν = 1486.6 eV). The cross-section of CoPc@BC after adsorption or catalytic oxidation was obtained by freezing the water-washed sample in liquid nitrogen and cracking with a blade. The binding energy peaks of the XPS spectra were calibrated by placing the principal C1s binding energy peak at 284.6 eV. 

### 2.4. Adsorption and Catalytic Oxidation Decoloration

**Figure 2:**
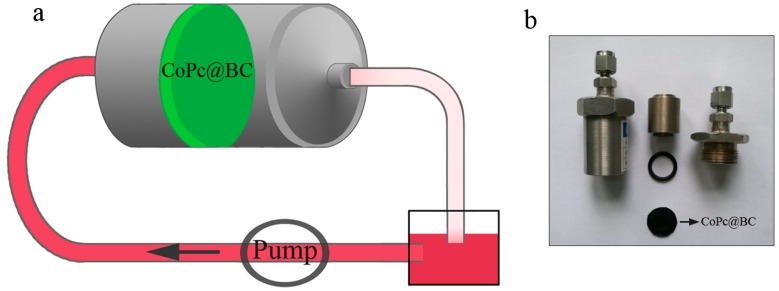
(**a**) Schematic representation of the CoPc@BC-based catalytic membrane reactor (CMR); (**b**) Optical image of the CoPc@BC-based CMR.

A CoPc@BC-based CMR was assembled for the decoloration of dye wastewater, and the experimental setup of the CMR is shown in Figure 2. In a typical adsorption experiment, the initial amount or concentration of a reaction mixture was controlled as follows: (a) 20 mm diameter and 14 ± 1 μm thick (ca. 1.60 mg) of CoPc@BC (430 ± 13 μmol/g); and (b) 10 mL of reactive red X-3B solution (100 μmol/L, 50 °C). The pH value of the dye solution was adjusted with H_2_SO_4_ or NaOH. The adsorption was investigated by the concentration of reactive red X-3B, which is proportional to its absorbance at 539 nm, as monitored by a UV-VIS absorption spectrometer UV-2450. The concentration change of reactive red X-3B was express as the change of C/C_0_ value, where C_0_ is the initial concentration of the dye, and C is the residual concentration of the dye. 

For the initiation of the catalytic oxidation process, a required amount of H_2_O_2_ was added into the above mentioned adsorption system. The measurement method for catalytic oxidation decoloration is the same as that of adsorption process, the decoloration rate of CoPc@BC (CoPc loading: 430 ± 13 μmol/g) was calculated using Equation (2):

(2) $$\mathrm{Decoloration}\;\mathrm{rate}\;(\mu mol\cdot \min^{-1}\cdot g^{-1})=\frac{100\;\mu mol/L\times 10\times 10^{-3}\;L\times 90\%}{t\;\min\times 1.60\times 10^{-3}\;g\times 430\times 10^{-6}\;mol/g\times 630\;g/mol}$$

where *t* is the time for decoloration of 90% of reactive red X-3B. EPR signal of radical spin-trapped by DMPO was detected with a Bruker-A300 X-band EPR spectrometer (Bruker, Karlsruhe, Germany). For every run in the cyclic utilizations, CoPc@BC was taken out, rinsed with ultrapure water, and dried at 25 °C for 12 h under vacuum. 

## 3. Results and Discussion

### 3.1. Characterization

Treating the cellulose nanofibers with NaIO_4_ is a well-established method for the generation of aldehyde groups, which can be potentially exploited as the binding sites for the immobilization of functional molecules [35,36]. Herein, the CoPc@BC nanocomposite was prepared by the covalent reaction of the amino groups on CoPc with the aldehyde groups on the oxidized BC. The results of the characterization study of the functionalization processes were given in our previous study and were used in the present study whenever it was necessary [16]. FESEM shows that the total nanofiber structure of the CoPc@BC nanocomposite was well maintained after functionalization (Figure S1, Supplementary Materials). ATR/FT-IR spectroscopy was adopted to monitor the progress of the surface modification reactions [16], as was shown in Figure S2. The strong absorption in the range of 3200–3500 cm^−1^ attributed to the vibration of the hydroxyl groups in the biosynthesized BC (Figure S2a). For the oxidized BC, the observation of a new characteristic peak at ~1650 cm^−1^ verified the success of oxidation process and the formation of the aldehyde groups on the surface of BC (Figure S2b). The expected appearance of a new absorption peak at ~1610 cm^−1^ (ν_C=N_ of CoPc) and the significant decrease of the characteristic peak at ~1650 cm^−1^ confirmed the successful reaction of the aldehyde groups and the immobilization of the CoPc molecules onto the BC (Figure S2c). 

**Figure 3:**
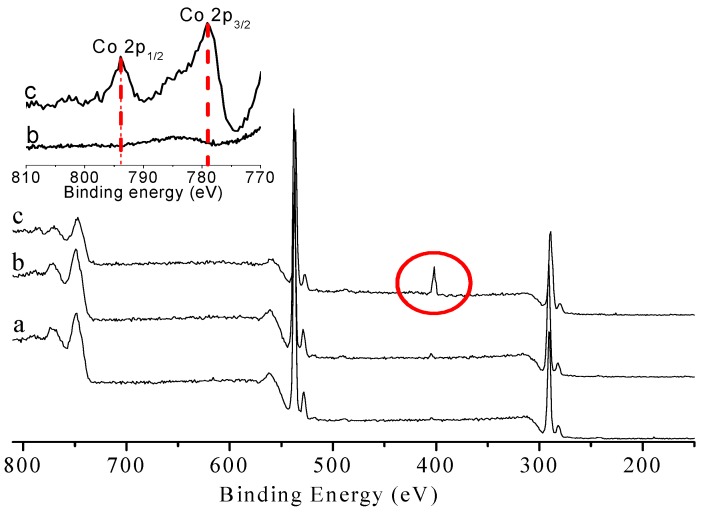
XPS spectra of (**a**) BC; (**b**) oxidized BC; and (**c**) CoPc@BC. The window included shows, in detail, the Co region.

XPS was also performed to analyze the chemical composition of the BC surface during the series of modification [16]. For the as-prepared BC (Figure 3a), the two characteristic peaks at 284.6 eV and 533 eV were ascribed to the binding energy of C1s and O1s, respectively. The oxidation process did not significantly change the XPS spectrum of the BC membrane (Figure 3b). Upon the immobilization of CoPc molecules onto the oxidized BC, an additional new peak at a binding energy of ca. 400 eV was observed (Figure 3c), which was the characteristic peak of N_1s_. Furthermore, two new signals at binding energy of 779.1 eV and 793.9 eV were also detected (Figure 3, inset), which were assigned to the characteristic peaks of Co 2p_3/2_ and Co 2p_1/2_, respectively. These results provide further evidence for the immobilization of CoPc onto BC and the successful preparation of CoPc@BC. 

**Figure 4:**
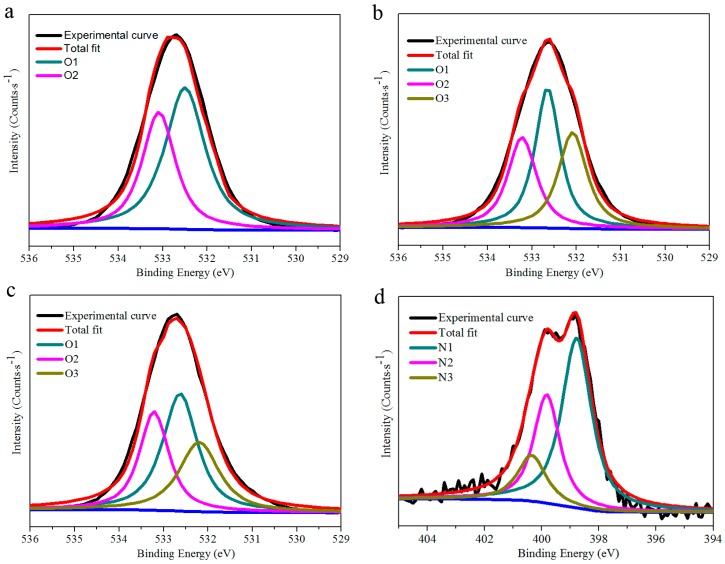
Curve fit of (**a**) O1s peaks of BC; (**b**) O1s peaks of oxidized BC; (**c**) O1s peaks of CoPc@BC; and (**d**) N1s peaks of CoPc@BC.

The high-resolution O1s peaks and N1s peaks in the XPS spectra of nanofiber mats gives detailed information on O and N chemistry during the functionalization processes (Figure 4). For pure BC, the O1s peak centered at 532.5 eV was ascribed to C-OH (O1, BC of Figure 1 and Figure 4a), and the O1s peak occurring at 533.1 eV was due to C–O–C (O2, BC of Figure 1 and Figure 4a) [37]. For oxidized BC, besides the detection of O1 and O2, the new O1s peak present at 532.1 eV was assigned to C=O (O3, oxidized BC of Figure 1 and Figure 4b), implying the successful formation of aldehyde groups. The proportion of C=O peak is smaller in CoPc@BC than that of oxidized BC, indicating that a part of aldehyde groups were reacted with CoPc (O3, Figure 4c). The remaining existence of aldehyde groups was likely due to the steric hindrance effect for the immobilization and the incompleteness character of macromolecular reaction. 

Furthermore, the N1s peaks give detailed information of the bonding environment of CoPc@BC (Figure 4d). The N1s peaks present at 398.8 eV and 399.8 eV were described to aza-bridging nitrogen, pyrrole nitrogen (N1, CoPc@BC of Figure 1 and Figure 4d), and nitrogen of peripheral amino groups (N2, CoPc@BC of Figure 1 and Figure 4d), respectively [38,39]. In addition, the peak at 400.5 eV was assigned to the nitrogen of –C=N– (N3, CoPc@BC of Figure 1 and Figure 4d), which was the reaction product of aldehyde groups of the oxidized BC and amino groups of CoPc. These results provided clear evidence for successful modification and covalent immobilization of CoPc on BC. 

### 3.2. Optimization of Immobilization Conditions

For possible industrial application, high CoPc loading is desired to reduce the size of reactor and enhance the efficiency of catalytic reaction; hence, the content of aldehyde groups should be maximized. In this study, three parameters (NaIO_4_ concentration, oxidation temperature, and oxidation time) describing different oxidation conditions with the corresponding CoPc loading were carefully studied. 

**Figure 5:**
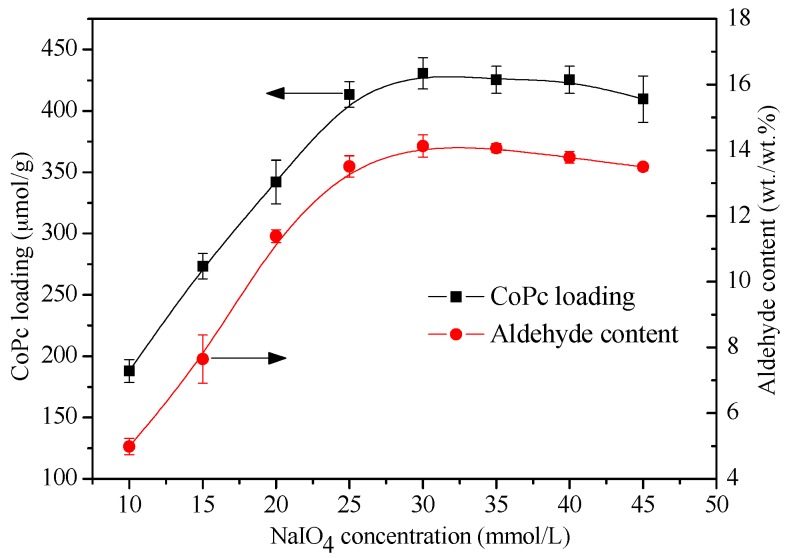
Effect of NaIO_4_ concentration on CoPc loading of CoPc@BC (filled square) and aldehyde content of BC (filled circle), T = 30 °C, reaction time = 8 h.

The effect of NaIO_4_ concentration on the CoPc loading was assayed within the concentration range from 10 mmol/L to 45 mmol/L, and the typical result is shown in Figure 5. Both the aldehyde groups’ content and the CoPc loading increased rapidly with NaIO_4_ concentration. To provide more binding sites for CoPc immobilization, a sufficient amount of oxidizer (NaIO_4_) must be applied to maximize the yield of the aldehyde groups. The CoPc loading was less than 200 μmol/g with 10 mmol/L NaIO_4_. When NaIO_4_ concentration increased to 30 mmol/L, the aldehyde groups’ content of the oxidized BC increased from 5 to 14 wt./wt.% and, accordingly, the CoPc loading was as high as 430 μmol/g. However, a further increase of NaIO_4_ concentration results in the slow decrease of CoPc loading. This phenomenon can be attributed to two reasons. Firstly, under a very high concentration of NaIO_4_, a portion of the formed aldehyde groups were likely to further oxidize to carboxyl groups [40], reducing the binding sites which can readily react with CoPc, thus leading to a slightly lower level of CoPc loading. This result is in accordance with the gradual decrease of the aldehyde groups’ content under very high NaIO_4_ concentrations (Figure 5). The second explanation is related to the gradual degradation of cellulose chains with the high concentration of NaIO_4_ [36], which is quite possible for the very large surface to volume ratio of BC. 

An increased temperature range from 10 °C to 45 °C was applied to study the effect of oxidation temperature on the CoPc loading. As shown in Figure S3, both the aldehyde content and the CoPc loading increased rapidly with the oxidation temperature, CoPc@BC with ca. 430 μmol/g of CoPc was obtained at 30 °C. Above this temperature, the CoPc loading of BC decreased gradually. A similar result was also found for the investigation of the influence of oxidation time on the aldehyde content and the CoPc loading (Figure S4). These results can also be explained by the reason discussed above. With the increase of oxidation temperature and oxidation time, more aldehyde groups were formed correspondingly on BC, that is, more binding sites were generated for CoPc immobilization. However, one should note that the transformation of the aldehyde groups to the carboxyl groups was possible with very high oxidation temperature and further elongation of oxidation time.

Therefore, a NaIO_4_ concentration of 30 mmol/L, an oxidation temperature of 30 °C, as well as an oxidation time of 8 h was chosen for the oxidation of BC, and the resulting oxidized BC contains 14.13% (wt./wt.) of the aldehyde groups, which is slightly lower than the theoretical maximum content of 18.1% (wt./wt.). The relatively low yield of aldehyde groups can be ascribed to the difficulty of heterogeneous reaction and the incompletion of macromolecular reaction. Under the optimal oxidation conditions, a CoPc@BC with 430 ± 13 μmol/g of CoPc was prepared, indicating the high reliability of this technique for the immobilization of CoPc. 

### 3.3. Adsorption of Dye Molecules on CoPc@BC

**Figure 6:**
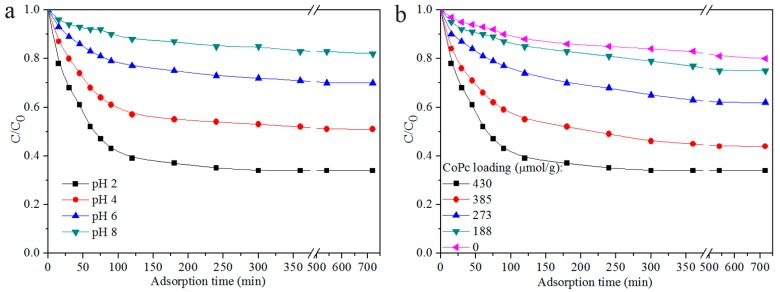
Concentration changes of reactive red X-3B (initial concentration: 1 × 10^−4^ mol/L, T = 50 °C) with CoPc@BC (1.60 mg) adsorption: (**a**) effect of pH; and (**b**) the effect of CoPc loading.

The prepared CoPc@BC nanocomposite was aimed as a functional nanomaterial for the catalytic oxidation of organic pollutants, with reactive red X-3B dye wastewater as a model contaminant. For a heterogeneous catalyst, the catalytic reaction mainly consists of two parts: the adsorption of the substrate onto the solid and the following degradation reaction of the adsorbed substrate [41]. Thus, the interaction between the CoPc@BC catalyst and the dye substrate is indispensable for the efficient catalytic oxidation reaction. Both the surface character of CoPc@BC and the property of the dye molecules have a great effect on this interaction. Herein, by monitoring the concentration change of reactive red X-3B in the bulk solution, the influence of pH value on the adsorption behavior of CoPc@BC was firstly investigated (Figure 6a). The decrease of the dye concentration demonstrates the gradual adsorption of dye molecules onto the heterogeneous catalyst. When the adsorption reached a dynamic equilibrium, the dye concentration remains at a certain level and does not decrease with a further elongation of time. It was found that both the adsorption rate and the saturated adsorption amount were significantly increased with the decline of pH. The saturated adsorption amount was 66% at pH 2, compared with 50% at pH 4 and 30% at pH 6. The highly-saturated adsorption amount of CoPc@BC under acidic conditions is derived from the strong electrostatic interaction between the CoPc molecules on the nanocomposite and the dye molecules. The amino groups of CoPc molecules can function as an “antenna” to accept a proton from the acidic solution, endowing the surface of CoPc@BC with positive charges, which can be preferably adsorbed by the anionic dye molecules to a considerable extent via electrostatic interaction with the sulfonic groups of the reactive red X-3B. 

The influence of CoPc loading on the adsorption process was also investigated (Figure 6b). It is obvious that the increase of CoPc loading can efficiently increase both the adsorption rate and the saturated adsorption amount of dye molecules. The increased “antenna” CoPc molecules reasonably increased the proton accepting ability of CoPc@BC nanocomposite, which results in its enhanced dye adsorption ability. Thanks to the very large surface-to-volume ratio of BC, the CoPc loading was much higher comparing with that of traditional solid supports, thus, the saturated adsorption amount of CoPc@BC nanocomposite is, accordingly, very high. 

**Figure 7:**
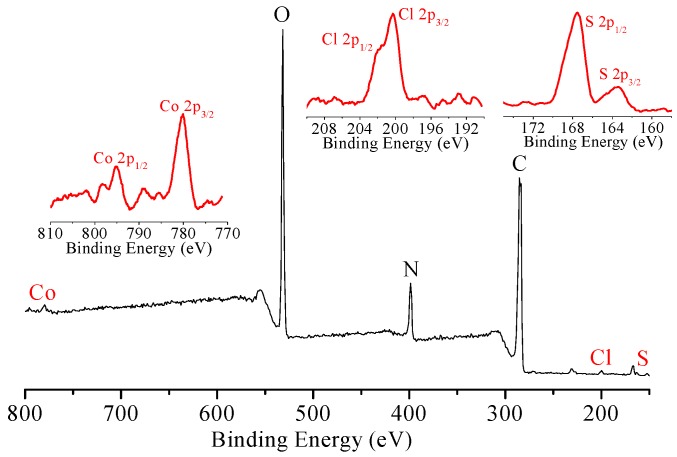
XPS of the cross-section of CoPc@BC after dye adsorption. **Left** inset: detail of the Co region, **middle** inset: detail of the Cl region; and **right** inset: detail of the S region.

The composition of CoPc@BC, both in cross-section and on the surface after dye adsorption, was studied with XPS, and the results are shown in Figure 7 and Figure S5, respectively. Compared with the original CoPc@BC (Figure 3c), two new signals at binding energies of 163.5 eV and 167.9 eV were assigned to the characteristic peaks of S 2p_3/2_ and S 2p_1/2_, respectively (Figure 7 and Figure S5, right inset). The new signals at binding energies of 200 eV and 202 eV were ascribed to the characteristic peaks of Cl 2p_3/2_ and Cl 2p_1/2_, respectively (Figure 7 and Figure S5, middle inset). The detection of these two elements implies that the adsorption occurs not only on the surface, but also in the deep interior of CoPc@BC. Thanks to the highly three-dimensional porous network structure, the contact between the CoPc catalyst and dye molecules is more effective than other supports, which is quite important for the subsequent catalytic oxidation. 

### 3.4. Catalytic Oxidation Studies under Various Operational Conditions

**Figure 8:**
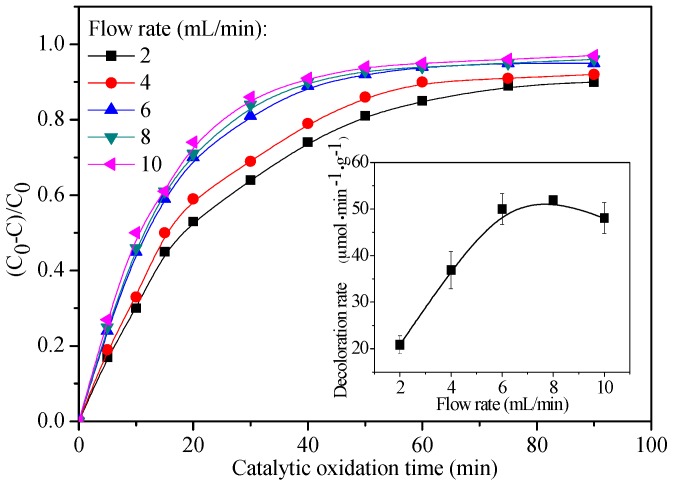
Typical concentration changes of reactive red X-3B (T = 50 °C, pH = 2) with CoPc@BC (1.60 mg) and H_2_O_2_ (10 mmol/L) under various flow rates. Inset: Effect of flow rate on the decoloration rate of reactive red X-3B.

The performance of CMR assembled with CoPc@BC is evaluated in terms of the catalytic oxidation efficiency of reactive red X-3B, with H_2_O_2_ as an oxidant. To explicitly show how CoPc@BC affects the catalytic oxidation reaction, blank experiments were performed under H_2_O_2_ oxidation without the addition of the CoPc@BC catalyst. Almost no color change can be detected after 4 h with the existence of H_2_O_2_ (data not shown), indicating that CoPc@BC is indispensible for the catalytic oxidation reaction. Figure 8 shows the decoloration of the dye solution with time under various flow rates. The rapid decrease of dye concentration indicates that the catalytic oxidation reaction occurring during the operation, and the decoloration rate depends strongly on the dye solution flow rate (Figure 8, inset). The decoloration rate was ca. 50 μmol⋅min^−1^⋅g^−1^ when the dye solution flow rate was set to 6 mL/min, comparing with ca. 20 μmol⋅min^−1^⋅g^−1^ at flow rate of 2 mL/min. A higher flow rate can effectively decrease the mass transfer resistance of dye molecules and H_2_O_2_ to the CoPc@BC nanocomposite, increasing both the contact opportunity and the contact frequency among the reactant dye molecules, H_2_O_2_, and the heterogeneous CoPc@BC catalyst. In this way, the catalytic oxidation rate of dye molecules was effectively increased. However, a further increase of the dye solution flow rate does not consistently result in the increase of the decoloration rate. This is possibly due to the shorter contact time between the CoPc@BC and the reactants at too high a flow rate, thus, the dye molecules cannot be sufficiently catalytic oxidized by CoPc@BC/H_2_O_2_ reaction system. In this regard, we chose 6 mL/min as the optimal dye solution flow rate. 

The effect of the reaction temperature on the catalytic oxidation performance of CMR is shown in Figure S6. Reactive red X-3B dye wastewater is able to be catalytically oxidized over a broad temperature range. The catalytic oxidation rate increased with the reaction temperature, while only a slight increase in the reaction rate can be achieved when the reaction temperature is higher than 50 °C. Higher temperatures cause a greater chance of self-decomposition of H_2_O_2_. Considering both the catalytic oxidation efficiency and the minimization of energy consumption, the optimal operational temperature was set to 50 °C. 

Figure S7 shows the decoloration rate as a function of H_2_O_2_ concentration. It is conceivable that a relatively higher catalytic oxidation rate could be obtained by increasing the concentration of the H_2_O_2_ oxidant during the reaction. The catalytic oxidation rate was ca. 50 μmol⋅min^−1^⋅g^−1^ with the addition of 10 mM H_2_O_2_, comparesd with ca. 20 μmol⋅min^−1^⋅g^−1^ when the H_2_O_2_ concentration is 6 mM. A slight reaction rate change was observed when the H_2_O_2_ concentration was higher than 10 mM. In this regard, we chose 10 mM H_2_O_2_ as the optimal oxidant concentration. 

Therefore, a flow rate of 6 mL/min, together with the reaction temperature of 50 °C and H_2_O_2_ concentration of 10 mM, were required for this high-performance CMR. Under these optimal operating conditions, the decoloration rate of the CMR was ca. 50 μmol⋅min^−1^⋅g^−1^. The catalytic oxidation efficiency of the present CMR is much higher compared with the related literature we reported previously [32,42]. 

**Figure 9:**
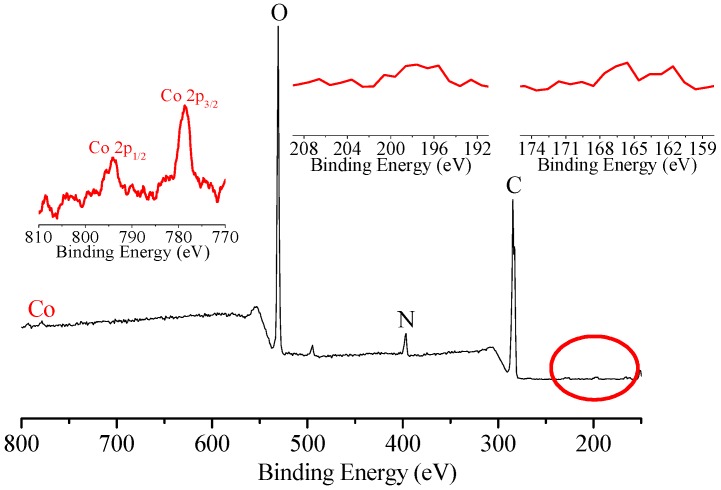
XPS of cross-section of CoPc@BC after catalytic oxidation. **Left** inset: detail of Co region, **middle** inset: detail of Cl region, **right** inset: detail of S region.

The composition of cross-section of CoPc@BC after catalytic oxidation was analyzed with XPS (Figure 9). The two peaks corresponding to Co 2p_3/2_ and Co 2p_1/2_ remains can be detected after reaction. However, almost no signal was observed in the range of binding energy between 210 eV and 160 eV (the position of characteristic peaks of S and Cl), which indirectly verified that the adsorbed dye molecules were successfully catalytically oxidized. A similar result was obtained for the XPS of the surface composition of CoPc@BC (data not shown). 

**Figure 10:**
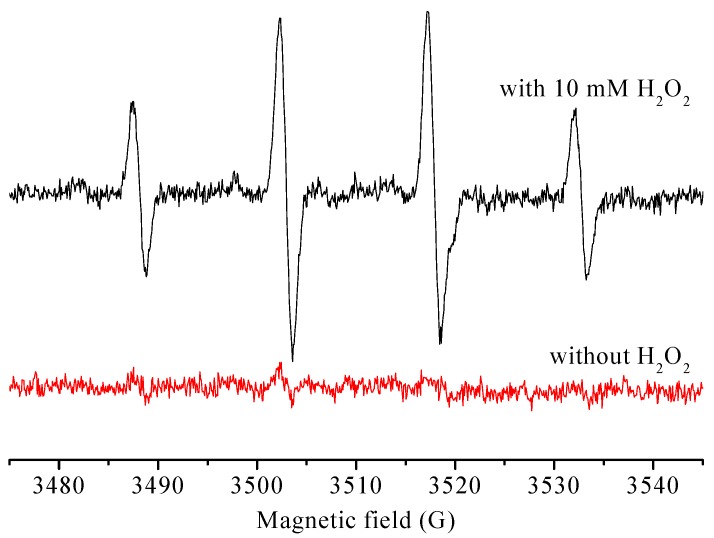
DMPO spin-trapping EPR spectra of reactive red X-3B dye solution (initial concentration: 1 × 10^−4^ mol/L, CoPc@BC: 1.60 mg, T = 50 °C).

The EPR spin-trapping technique was applied to monitor the formation of reactive species which are responsible for the catalytic oxidation of reactive red X-3B by CoPc@BC/H_2_O_2_ reaction system. As shown in Figure 10, no signal was observed without the addition of H_2_O_2_. However, obvious signals with four peaks were generated when 10 mM of H_2_O_2_ was added, which are the typical characteristic peaks of DMPO-OH adducts [43], indicating the involvement of highly-reactive ⋅OH during the catalytic oxidation process. 

In practical applications, the stability and the recyclability of the catalyst should be taken into account. The catalytic activity of CoPc@BC in cyclic utilizations was investigated, and no significant decrease of catalytic efficiency was found (Figure S8). The catalytic activity of CoPc@BC remained excellent after recycling five times. No CoPc absorbance peak (in the range of 600–800 nm) was detected during the reaction. These results indicate that the CoPc@BC/H_2_O_2_ reaction system is recyclable for consecutive catalytic oxidation of dye wastewater, which is desirable for industrial applications. 

**Figure 11:**
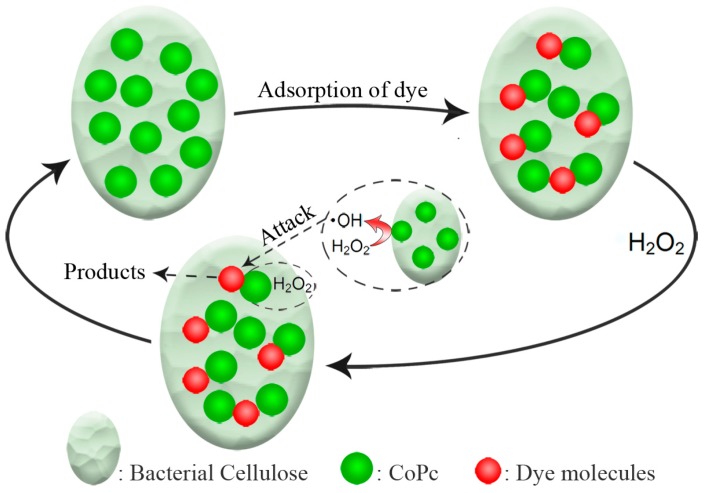
Conceptual representation of catalytic oxidation of reactive red X-3B with CoPc@BC/H_2_O_2_ reaction system.

On the basis of the results above, a proposed mechanism of catalytic oxidation of dye molecules with the CoPc@BC/H_2_O_2_ reaction system is presented in Figure 11. H_2_O_2_ was catalyzed by CoPc@BC to form highly-reactive hydroxyl radicals, and the produced ⋅OH will attack the adsorbed dye molecules which were “anchored” by the CoPc “antenna”. The high CoPc loading of CoPc@BC allows the easily adsorption of dye molecules, and the circulation system of CMR can effectively promote the formation of ⋅OH and the further catalytic oxidation of the adsorbed dye, thus, the catalytic efficiency was substantially improved. 

## 4. Conclusions

In the current research, we have systematically quantified the immobilization of CoPc onto BC for the fabrication of CoPc-immobilized BC nanocomposite, CoPc@BC. The optimal processing conditions are as follows: NaIO_4_ concentration of 30 mmol/L, an oxidation temperature of 30 °C, and an oxidation time of 8 h. The potential ability of CoPc@BC for the removal of dye wastewater was thoroughly investigated, the saturated adsorption amount of CoPc@BC increased with the decrease of pH and the increase of CoPc loading. A catalytic membrane reactor (CMR) assembling with CoPc@BC was designed and operated. Under optimal operating conditions of a dye solution flow rate of 6 mL/min, a reaction temperature of 50 °C, and an H_2_O_2_ concentration of 10 mmol/L, the decoloration rate of CMR was as high as 50 μmol⋅min^−1^⋅g^−1^. EPR spectral results revealed that ⋅OH is responsible for the catalytic oxidation of dye molecules. 

## Supplementary Materials

The following are available online at www.mdpi.com/1996-1944/10/7/846/s1. Figure S1. FESEM of (a) pure BC, and (b) CoPc@BC; Figure S2. ATR/FT-IR spectra of (a) BC, (b) oxidized BC, and (c) CoPc@BC; Figure S3. Effect of oxidation temperature on CoPc loading of CoPc@BC (filled square) and aldehyde content of BC (filled circle); Figure S4. Effect of oxidation time on CoPc loading of CoPc@BC (filled square) and aldehyde content of BC (filled circle); Figure S5. XPS of surface of CoPc@BC after dye adsorption. Left inset: detail of Co region, middle inset: detail of Cl region, right inset: detail of S region; Figure S6. Effect of reaction temperature on decoloration rate of reactive red X-3B (flow rate: 6 mL/min, H_2_O_2_ concentration: 10 mmol/L); Figure S7. Effect of initial H_2_O_2_ concentration on decoloration rate of reactive red X-3B (flow rate: 6 mL/min, T = 50 °C); Figure S8. Repetitive catalytic oxidation of reactive red X-3B (initial concentration: 1 × 10^−4^ mol/L, CoPc@BC: 1.60 mg, flow rate: 6 mL/min, H_2_O_2_ concentration: 10 mmol/L, T = 50 °C) for 60 min.
