# Supplementary material for: Quantitative Immobilization of Phthalocyanine onto Bacterial Cellulose for Construction of a High-Performance Catalytic Membrane Reactor
Source: Materials (Basel). 2017 Jul 24;10(7):846. doi: 10.3390/ma10070846 (PMC5551888; doi:10.3390/ma10070846)
Supplement: Supplementary file 1 [file materials-10-00846-s001.pdf]

materials

MDPI

Article

Quantitative Immobilization of Phthalocyanine onto Bacterial Cellulose for Construction of High Performance Catalytic Membrane Reactor

Shiliang Chen*, Qiaoling Teng

Qianjiang College, Hangzhou Normal University, Hangzhou 310012, China; tengql1996@sina.com (Q.T.)
* Correspondence: bruceblue@zju.edu.cn (S.C.); Tel.: +86 571 28861372

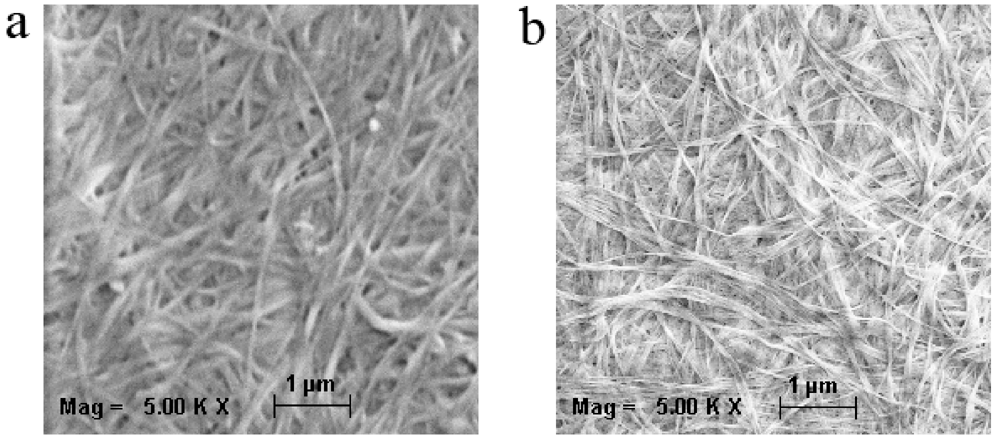

Figure S1. FESEM of (**a**) pure BC, and (**b**) CoPc@BC.

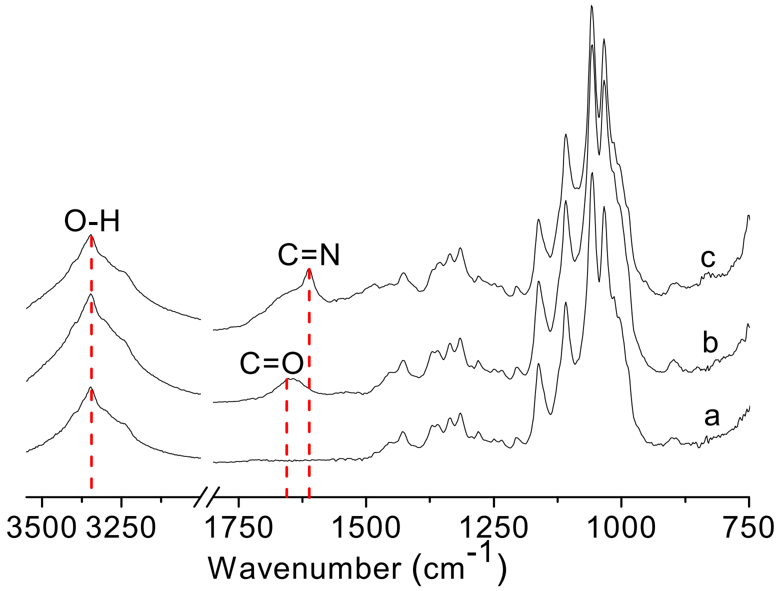

Figure S2. ATR/FT-IR spectra of (**a**) BC, (**b**) oxidized BC, and (**c**) CoPc@BC.

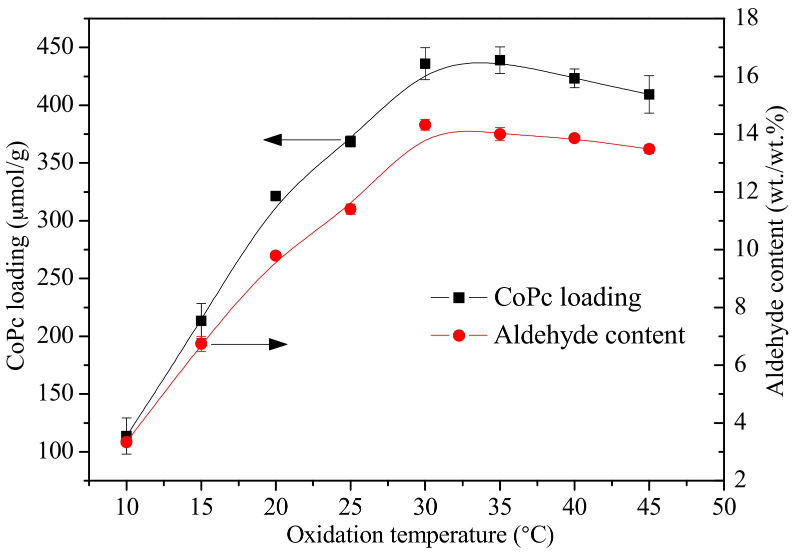

Figure S3. Effect of oxidation temperature on CoPc loading of CoPc@BC (filled square) and aldehyde content of BC (filled circle), [$NaIO_4$]=30 mmol/L, reaction time=8 h.

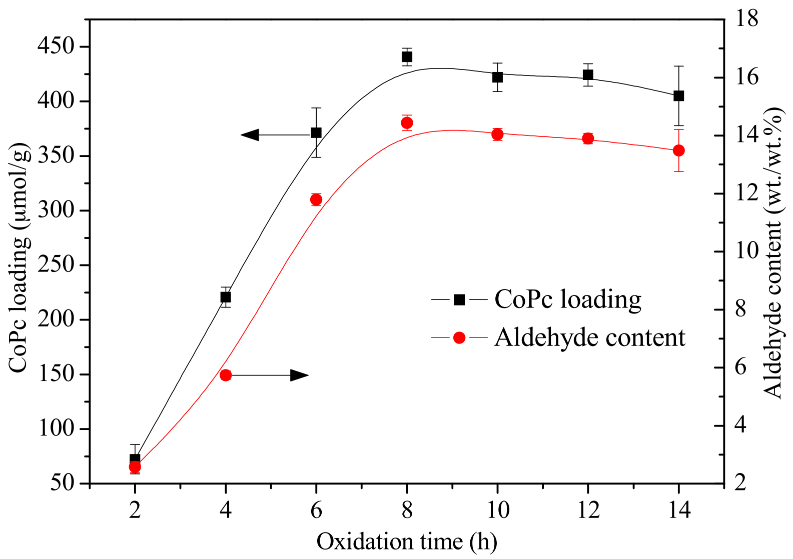

Figure S4. Effect of oxidation time on CoPc loading of CoPc@BC (filled square) and aldehyde content of BC (filled circle), [$NaIO_4$]=30 mmol/L, T=30 °C.

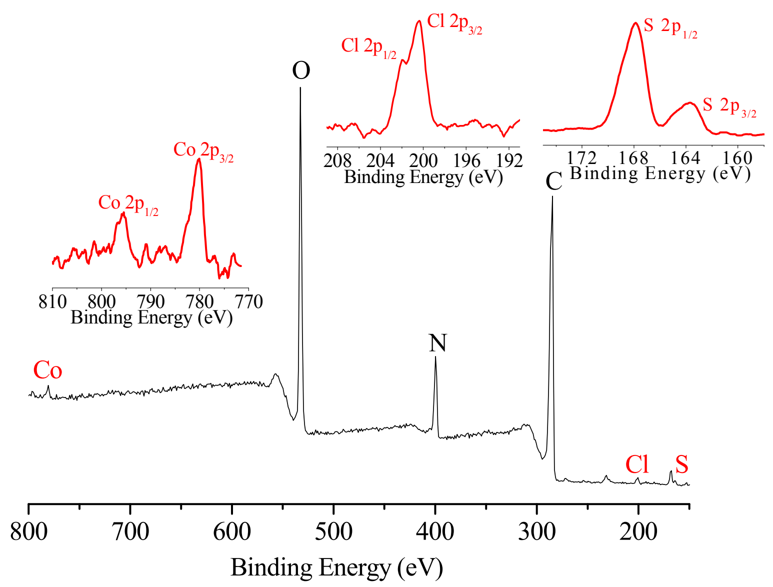

Figure S5. XPS of surface of CoPc@BC after dye adsorption. Left inset: detail of Co region, middle inset: detail of Cl region, right inset: detail of S region.

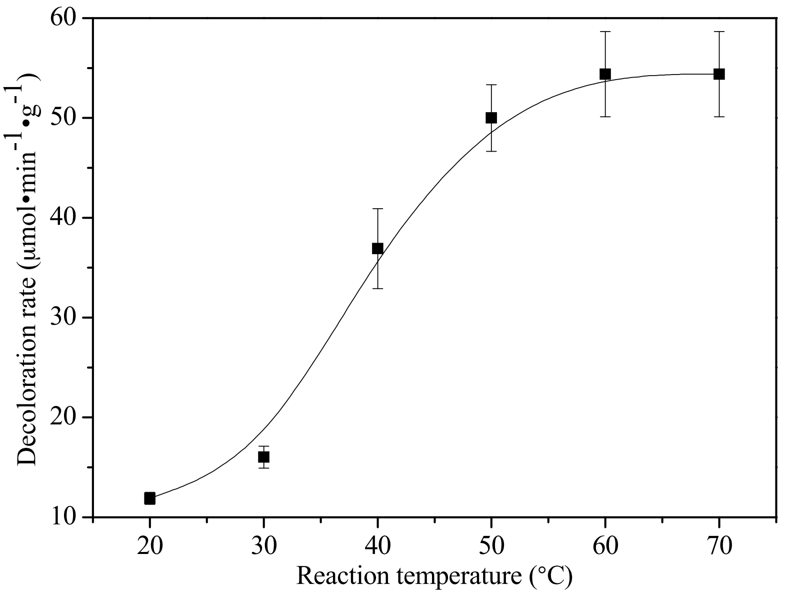

Figure S6. Effect of reaction temperature on decoloration rate of reactive red X-3B (flow rate: 6 mL/min, H_2O_2 concentration: 10 mmol/L).

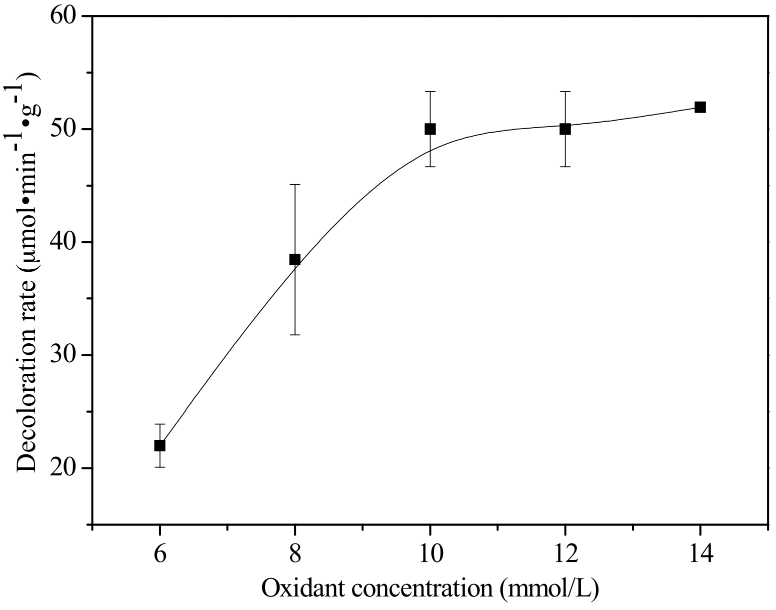

Figure S7. Effect of initial H_2O_2 concentration on decoloration rate of reactive red X-3B (flow rate: 6 mL/min, T=50 °C).

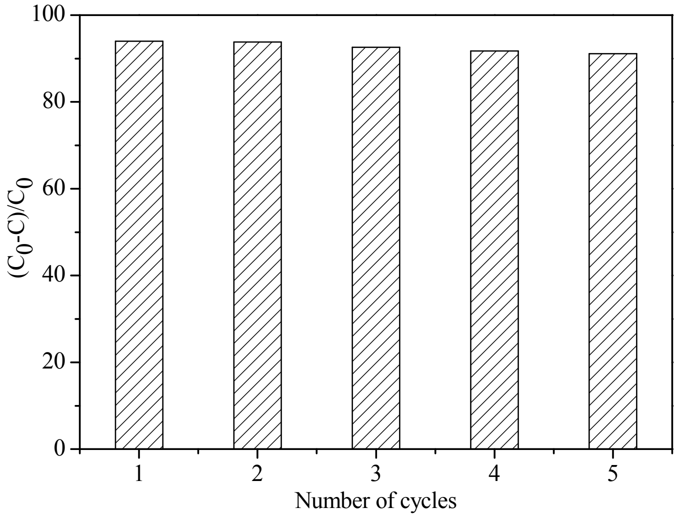

Figure S8. Repetitive catalytic oxidation of reactive red X-3B (initial concentration: 1 × 10^{-4} mol/L, CoPc@BC: 1.60 mg, flow rate: 6 mL/min, H_2O_2 concentration: 10 mmol/L, T=50 °C) for 60 min.
